# Monthly Summary

**Published:** 1874-06

**Authors:** 


### MONTHLY SUMMARY.

---

*Carbolic Acid in Carbuncle.*—Mr. Peter Eade describes a case of carbuncle treated by carbolic acid, in the *Lancet.* He says: Into the centre of the two holes which had formed I pressed with a probe some threads of lint soaked in a strong solution of carbolic acid in oil (one part to four,) and I also laid a piece of lint wet with the same over their apertures, so as to supplement the small quantity which the shallow sinuses would contain. A little smarting was complained of, but the application was repeated after a few hours, and again the following day. Almost at the end of twenty-four hours it could be perceived that a check had taken place in the morbid process, but by the next day it was plainly evident that the inflammation and induration were really beginning to subside. The carbolized lint was still carefully and scrupulously thrust to the very bottom of the small holes, and from this time no further spread of the disease took place, but, on the contrary, there was a rapid subsidence of the œdema, and in two or three days more, little remained but some diffused swelling of the lower lip, some tender induration at and around the seat of the original pimple, and the ragged discharging opening which had formed at the site of the primary festers. The disease was therefore stayed, and in a few days more the patient was convalescent. I have now used carbolic acid in this way in several cases of carbuncle; and in all of them its application has been followed by a uniform and immediate check to its increase, and a speedy amelioration of the local conditions. When it has been applied early, it has plainly gone far to abort the disease; and when it has been commenced later, wherever it could be brought into contact with the inflamed and hardened tissue, there at least no further spreading has taken place, whilst swelling and tension have diminished, and dirty suppurating slough has quickly given place to florid healthy granulation.

And from my observation of its action, I entertain no doubt that, if it could be brought sufficiently early into contact with the spreading disease, it would be quite competent to prevent its extension beyond the degree to which it had already advanced. Unfortunately, the acid appears to have little or no influence when applied over the unbroken skin ; but directly it can be brought into contact with the diseased mass, either by being inserted into the sieve-like holes, or by being applied to it after being laid open by incision, its beneficial action becomes at once manifest.—*Medical and Surgical Reporter.*

---

*Disease-Destroying Tree.*—M. GIMBERT, who has been long engaged in collecting evidence concerning the Australian tree *Eucalyptus globulus*, the growth of which is surprisingly rapid, attaining, besides, gigantic dimensions, has addressed an interesting communication to the Academy of Science. This plant, it now appears, possesses an extraordinary power of destroying miasmatic influence in fever stricken districts. It has the singular property of absorbing ten times its weight of water from the soil, and of emitting antiseptic camphorous effluvia. When sown in marshy ground it will dry up in a very short time. The English were the first to try it at the Cape, and within two or three years they completely changed the climatic condition of the unhealthy parts of the colony. A few years later its plantation was undertaken on a large scale in various parts of Algeria. At Pardock, twenty miles from Algiers, a farm situated on the banks of the Hamyze was noted for its extremely pestilential air. In the spring of 1867 about 13,000 of the eucalyptus were planted there. In July of the same year —the time when the fever season used to set in—not a single case occurred ; yet the trees were not more than nine feet high. Since then complete immunity from fever has been maintained. In the neighborhood of Constantine the farm of Ben Machydlin was equally in bad repute. It was covered with marshes both in winter and summer. In five years the whole ground was dried up by 14,000 of these trees, and farmers and children enjoy excellent health. At the factory of the Glue de Constantine, in three years a plantation of eucalyptus has transformed twelve acres of marshy soil into a magnificent park, whence fever has completely disappeared. In the Island of Cuba this and all other paludal diseases are fast disappearing from all the unhealthy districts where this tree has been introduced. A station-house at one of the ends of a railway-viaduct in the department of the Var was so pestilential that the officials could not be kept there longer than a year. Forty of these trees were planted, and it is now as healthy as any other place on the line. We have no information as to whether this beneficent tree will grow in other than hot climates. We hope that experiments will be made to determine this point. It would be a good thing to introduce it on the West Coast of Africa.—*London Med. Times.*

---

*Extraordinary Malpractice.*—The following account is sent us by a correspondent in Baltimore. It is needless to say that the two physicians were both irregular practitioners. A young lady had been ill some time, and her sapient advisers decided she had Bright's disease, for which they prescribed a *turpentine vapor bath.* The bath was administered under the supervision of the doctors (?) in the following manner :—The patient having had the clothing removed, and been enveloped in blankets, was placed upon a chair with a hole in the seat. Beneath her was suspended a tin vessel containing the turpentine, and under it was placed a spirit lamp. The chair was covered with blankets and the lamp lighted. In a few minutes the patient sprang from the chair, exclaiming that she was on fire, simultaneously an explosion occurred, disseminating the ignited fluid about the chamber. The lady was terribly burned in the regions of the nates and thighs. She suffered excruciating agony during the three succeeding days, when death relieved her. The authors of her sufferings stated that they regretted exceedingly the unfortunate accident; (?) that it was an experiment they had never before tried, but from which they had hoped good results; that the small quantity of vapor, with which she had come in contact, had *already greatly benefited her kidneys*; and that the accident did not materially affect the result, since her case was hopeless. —*Medical and Surgical Reporter.*

---

*Chloral Hydrate and Camphor as a Local Application in Neuralgia.*—It is stated that the intimate mixture of equal parts of chloral hydrate and camphor will produce a clear fluid which is of the greatest value as a local application in neuralgia. Mr. LENOX BROWNE relates (*Brit. Med. Jour.*, March 7, 1874) that he has employed it and induced professional friends to do so, and that in every case it afforded great, and in some instantaneous relief. "Its success does not appear," he says, "to be at all dependent on the nerve affected, it being equally efficacious in neuralgia of the sciatic as of the trigeminus. I have found it of the greatest service in neuralgia of the larynx, and in relieving spasmodic cough of a nervous or hysterical character." It is only necessary to paint the mixture lightly over the painful part, and to allow it to dry. It never blisters, though it may occasion a tingling sensation of the skin. He has found it also an excellent application for toothache.—*Med. News and Library.*

*Poisoning by Cantharidal Collodion.*—Dr. Ernst Schwerin, of Berlin, reports a case (*Berliner Klinische Wochenschrift*) of poisoning with cantharidal collodion. The patient, a woman aged twenty-three years, swallowed, through mistake, fifteen drops of the preparation. After about an hour had elasped she was attacked with cramps in the lower part of the abdomen, for which previously to sending for a physician, numerous household remedies were used. The doctor upon his arrival found the patient running about the room, with the arms crossed upon the abdomen, stopping after every few steps to void a few drops of urine, the passage of which was attended with intense pain. At times she fell into a species of catalepsy. The pulse was small and of moderate frequency. For some days albumen was found in the urine. Under treatment, she at the end of a few days was entirely recovered. It is interesting to notice that the sexual passion was not at all excited by the drug; and this goes to confirm the opinion of later observers, that the older physicians were mistaken in attributing aphrodisiac qualities to it. —*Phila. Med. Times.*

---

*Remedy for Tooth-ache.*—Dr. Q. C. Smith, of Cloverdale, writes us:—Having spent a number of years of my professional life in the couutry, where no dentist was near, and therefore being often called to prescribe for tooth-ache, I tried many combinations of drugs, and many published formulas, with more or less satisfaction to myself and patients. But several years since I hit upon the following combination, which I have found better than any I have ever used, and it will give relief in almost all cases where such a remedy is applicable:

℞. Carbolic acid, saturated solution; hydrate of chloral, saturated solution; camphorated tinct. of opium; fluid extract of aconite, of each an ounce; oil of peppermint, ½ oz. Mix. Apply by saturating a pledget of cotton, (or preferably a small piece of sponge) and pack closely into the cavity of the decayed tooth.—*Pacific Med. and Surg. Journal.*

---

*A New Sign of Death.*—M. Bouchut stated, at the Academic des Sciences, that at the moment of death gases which are normally imprisoned in the venous blood are disengaged, forming a pneumatosis of the veins. The pneumatosis of the veins of the retina is easily appreciable by the ophthalmoscope, and constitutes an immediate and certain sign of death. This pneumatosis is indicated at the moment of death by the interruption of the column of blood in these veins, a phenomenon similar to that which is observed in the interrupted column of a thermometer with colored alcohol.—*Med. and Surg. Reporter.*

*A Dentist Fined.*—An action against a dentist for pulling the wrong teeth came before a court in New Hampshire, the other day. The plaintiff, being unavoidably absent by reason of having died, her interests were represented by her husband, who asserted that his wife gave the dentist special directions to pull certain teeth, and not to molest certain other teeth ; but he made a clean sweep of one jaw, and was rapidily harvesting the crop in the other, when the patient recovered from the anæsthetic and shut her mouth. The suit was for $5,000 damages, but the jury considering that it might have been a mere misunderstanding on the dentist's part, and that the plaintiff was dead, thought $20 about right.—*Druggist's Circular.*

---

*Successful Case of Transfusion.*—Prof. Liedeshorf, of Vienna, reports, (*Allgemeine Medicinnische Central-Zeitung*, Feb. 18, 1874) a successful case of transfusion resorted to in the case of a man, aged 23 years, who, in consequence of heavy pecuniary losses, had become greatly depressed, and had finally fallen into a cataleptic condition, which had lasted three weeks. Reflex sensibility was greatly impared, and no relief was experienced from the application of electricity. As a last resort, recourse was had to transfusion, and three ounces of blood were therefore injected. The temperature at once rose from 36° to 39·5°, and the pulse from 45 to 80. The patient began shortly after to speak ; the cataleptic symptoms gradually disappeared, and complete recovery rapidily ensued.—*Boston Med. and Surg. Jour.*

---

*Monstrosity.*—Dr. Balle presented the other day to the *Faculte de Medecine*, a young girl aged fourteen, named Blanche Dumas, from Issondun, whose body, from the waist downwards, is double —the two parts acting independently of each other. The two legs she uses for walking belong each to a different trunk, while a third one is quite insensible to pain. Her health is good.—*Lancet.*

---

*A New Dental Hospital.*—The new dental Hospital, situated in Leicester Square, London, was formally opened upon the 2d of March, upon which occasion an address was delivered by Edward Sercombe, Esq., the President of the Odontological Society.

---

*Death from Chloroform and Hemorrhage.*—Mrs. George W. Wilson, in Chester, Pennsylvania, took chloroform a few weeks ago, and had fifteen teeth extracted. She died in a few minutes under the combined influence of hemorrhage and chloroform.

